# Examination of malaria service utilization and service provision: an analysis of DHS and SPA data from Malawi, Senegal, and Tanzania

**DOI:** 10.1186/s12936-019-2892-x

**Published:** 2019-07-29

**Authors:** Cameron Taylor, Annē Linn, Wenjuan Wang, Lia Florey, Hamdy Moussa

**Affiliations:** 1 The Demographic and Health Surveys (DHS) Program, ICF, 530 Gaither Road, Suite 500, Rockville, MD 20850 USA; 2 U.S. President’s Malaria Initiative, USAID, Washington, DC USA

**Keywords:** Malaria, Care seeking, Malaria-service readiness, Fever, Malawi, Tanzania, Senegal

## Abstract

**Background:**

Ensuring universal access to malaria diagnosis and treatment is a key component of Pillar 1 of the World Health Organization Global Technical Strategy for Malaria 2016–2030. To achieve this goal it is essential to know the types of facilities where the population seeks care as well as the malaria service readiness of these facilities in endemic countries.

**Methods:**

To investigate the utilization and provision of malaria services, data on the sources of advice or treatment in children under 5 years with fever from the household-based Demographic and Health Surveys (DHS) and on the components of malaria service readiness from the facility-based Service Provision Assessment (SPA) surveys were examined in Malawi, Senegal and Tanzania. Facilities categorized as malaria-service ready were those with: (1) personnel trained in either malaria rapid diagnostic testing (RDT), microscopy or case management/treatment of malaria in children; (2) national guidelines for the diagnosis and treatment of malaria; (3) diagnostic capacity (available RDT tests or microscopy equipment as well as staff trained in its use); and, (4) unexpired artemisinin-based combination therapy (ACT) available on the day of the survey.

**Results:**

In all three countries primary-level facilities (health centre/health post/health clinic) were the type of facility most used for care of febrile children. However, only 69% of these facilities in Senegal, 32% in Malawi and 19% in Tanzania were classified as malaria-service ready. Of the four components of malaria-service readiness in the facilities most frequented by febrile children, diagnostic capacity was the weakest area in all three countries, followed by trained personnel. All three countries performed well in the availability of ACT.

**Conclusions:**

This analysis highlights the need to improve the malaria-service readiness of facilities in all three countries. More effort should be focused on facilities that are commonly used for care of fever, especially in the areas of malaria diagnostic capacity and provider training. It is essential for policymakers to consider the malaria-service readiness of primary healthcare facilities when allocating resources. This is particularly important in limited-resource settings to ensure that the facilities most visited for care are properly equipped to provide diagnosis and treatment for malaria.

## Background

Ensuring universal access to malaria diagnosis and treatment is a key component of Pillar 1 of the World Health Organization (WHO) Global Technical Strategy for Malaria 2016–2030. To achieve universal access to malaria diagnosis and treatment, it is essential to ensure that malaria services are available and accessible to the population at risk. This requires an understanding of care-seeking practices by the at-risk population, as well as information about the conditions of the health facilities and the quality of services offered by health providers.

Prior research has shown that the affordability, acceptability and availability of services are strong determinants of a population’s care-seeking behaviour and, therefore, of service utilization [1–4]. While in many countries the national policy is to provide free treatment for children under 5 years of age, affordability remains a problem; financial constraints can be a major barrier for seeking care, although it is hard to quantify the indirect costs of seeking care, such as transport to the health facility or losing a day’s work to attend the facility [3, 5, 6]. Regarding acceptability of services, both health facility determinants and individual sociocultural factors affect a child’s caregiver’s decision to seek treatment. In some settings the characteristics of health services have been identified as barriers to care-seeking, including long waiting times, poor communication between providers and patients, and patient’s perceptions of effectiveness of treatment [7–10]. Individual factors include caregiver’s limited understanding of disease causation, symptomatology and severity of illness [11–14]. Availability of services at a facility can also affect the use of malaria services. Availability, or physical access, refers to whether health services and providers are supplied within reasonable reach of the individuals who need them, and whether the services meet people’s needs. Key influences on availability and access include facility hours of operation, drug shortages, geographic proximity, and ease of attending the facility [1, 3, 8, 15, 16].

The readiness of a facility to provide malaria services has multiple determinants. Managing authority of health facility (public or private) and the level of the health facility (primary, secondary) have been shown to affect malaria service readiness [17]. When comparing the malaria-service readiness of public *versus* private facilities, some studies have highlighted inadequacies of the formal public health sector to guarantee appropriate levels of drugs, as well as to provide training and guidelines to service providers [3, 18–21]. Private facilities face such challenges as inadequate oversite and monitoring, prescription of treatments that do not conform to the national policy, and less malaria rapid diagnostic test (RDT) availability compared with public facilities [22–26]. Malaria-service readiness is also associated with the level of the health facility. Historically, lower-level facilities (such as health posts) have not had sufficient diagnostic capacity because of lack of microscopes or specialized staff trained in microscopy [27]. However, the introduction of RDTs has removed a barrier for service readiness for lower-level health facilities, because these tests make parasitological diagnosis feasible at lower levels and lead to improved targeting of malaria treatment [28]. In addition, it has been widely shown that lower-level care providers, such as community health workers, when appropriately trained and supervised, are capable of delivering high-quality care for uncomplicated malaria cases with RDTs to diagnose cases [29] and administration of artemisinin-based combination therapy (ACT) medicines [30, 31].

This study investigates the utilization and provision of malaria services by examining data on the source of advice or treatment in children age under 5 years with fever from the household-based Demographic and Health Surveys (DHS) surveys and data on provision of care from the facility-based Service Provision Assessment (SPA) surveys from Malawi, Senegal and Tanzania. The results will allow for better prioritization of interventions that can improve service readiness in the facilities where most care seeking for malaria occurs.

## Study setting

### Malawi

In Malawi, primary-level care is delivered at health posts, dispensaries, maternity facilities, health centres, and community or rural hospitals, where primary and preventive care services are provided primarily by community health workers. Secondary-level care is provided at district hospitals, which serve as referral facilities that provide inpatient and outpatient services. Central hospitals that provide tertiary-level services serve as referral hospitals for the district hospitals. With the policy of decentralization, the health system gave authority to the district health management teams to deliver secondary and primary health services at the district level [32].

Malawi’s private-sector facilities include not-for-profit and for-profit facilities. Private not-for-profit facilities include independent church-affiliated facilities that belong to the Christian Health Association of Malawi (CHAM) and are primarily located in rural areas. These facilities charge user fees for care other than basic preventive and curative care services for which they are contracted by district health officers to provide free of charge. The private for-profit sector also operates health facilities and health programmes. The public sector supports private-sector facilities and faith-based organizations (FBOs) by providing staff training, public sector staffing for facilities, supervision, medicines, and vaccines [32].

### Senegal

The operations of Senegal’s health system are implemented at district level. Each of the 76 health districts has at least one health centre, which is a secondary health facility with a medical team that provides direct care and oversees the district’s prevention efforts. Health centres are attached to the district’s health posts, staffed by nurse or midwife, and generally serve as the first level of contact with the population. Health posts are responsible for a number of health huts, which have trained community health workers and the necessary structure and equipment for providing basic services that include the diagnosis and treatment of uncomplicated malaria. Since 2008, community case management of malaria can be provided by home care providers, who are lower-level community health workers who work from their own homes rather than in a health facility [33].

Tertiary facilities include hospital centres that provide specialized care. These hospitals, which are found at the regional, departmental or communal level, typically provide care coverage for approximately 150,000 residents. There are also seven national hospital centres in Dakar. The health system has a network of regional pharmacies that supply the facilities and care providers of the corresponding regions. These regional pharmacies receive supplies from the national pharmacy. In addition to public sector facilities, there are also a number of private clinics and health posts throughout the country [33].

### Tanzania

A central district government structure is the basis of Mainland Tanzania’s National Health System. In this structure, the Ministry of Health, Community Development, Gender, Elderly and Children (MoHCDEC), the President’s Office of Regional Administration, and the local government share the responsibility for the delivery of public health services. The central level develops policies and guidelines, which are implemented by the regional health teams. Health facilities include dispensaries, health centres and hospitals, which operate in a hierarchical manner. The health facilities may be administered by the government, faith-based voluntary organizations, or other private organizations, and may be parastatal in nature. Dispensaries at the primary level in villages serve between 6000 and 10,000 residents, secondary-level health centres serve between 50,000 and 80,000 in a ward, and district hospitals provide tertiary-level care for more than 250,000 residents and refer cases to the regional and consultant hospitals [34]. Pharmaceutical services are provided through public-sector and FBO health facilities, private pharmacies, and accredited drug dispensing outlets (ADDOs) [34].

In Zanzibar, health services are delivered through public, private (both for-profit and non-profit), and additional government health facilities administered by military or defence forces. In the hierarchical system in Zanzibar, primary level care is provided at primary health care units (PHCUs) and primary health care cottages (PHCCs) that also provide inpatient care and X-ray services. At secondary level, the district and regional hospitals provide care. Specialized hospitals provide care at tertiary level. Under the policy of devolution, the provision of public health services, particularly preventive services, has been delegated to district health management teams in Zanzibar’s 11 districts [34].

## Methods

The study examined data on use of malaria services for children under 5 years of age with fever from the household-based Demographic and Health Surveys (DHS) and data on service readiness from the facility-based Service Provision Assessment (SPA) surveys conducted in Malawi, Senegal and Tanzania. The analysis focused on these three countries because in each country the fieldwork for the DHS and the SPA was completed within 1 year of each other. Surveys analysed were the 2013–2014 Malawi SPA and 2015–2016 Malawi DHS, the 2016 Senegal Continuous SPA and 2016 Senegal Continuous DHS, and the 2014–2015 Tanzania SPA and 2015–2016 Tanzania DHS-MIS [34–39].

DHS surveys are nationally representative household surveys that provide data for a wide range of monitoring and impact evaluation indicators in the areas of population, health and nutrition. The DHS surveys have large sample sizes (usually between 5000 and 30,000 households). In malaria-endemic countries many DHS surveys include questions about malaria in both the Household Questionnaire and Woman’s Questionnaire. Responses to these questions generate data that can be used to assess progress in the core household malaria indicators that are detailed in the Roll Back Malaria Partnership to End Malaria (RBM) guidance document, *Household Survey Indicators for Malaria Control* [40].

This analysis uses the DHS survey indicator “proportion of children under age 5 with fever in the previous 2 weeks for whom advice or treatment was sought”. Although non-specific for malaria, fever in children is often a prompt for care seeking due to perceived risk of serious illness. In malaria-endemic countries, facilities receiving sick children should be following integrated management of childhood illness (IMCI) protocols, which include diagnosis and treatment of malaria.

SPA surveys are sample surveys of formal-sector health facilities. Typically, the SPA surveys collect data from 400 to 700 facilities selected from a comprehensive list of health facilities in a country (sampling frame), categorized by facility type, managing authority (public and non-public), and geographic region. The SPA surveys include four main questionnaires: (1) inventory questionnaire; (2) health worker or provider questionnaire; (3) observation protocols; and, (4) exit interview questionnaire. This analysis focuses on data from the inventory questionnaire and the health worker or provider questionnaire.

**Fig. 1:**
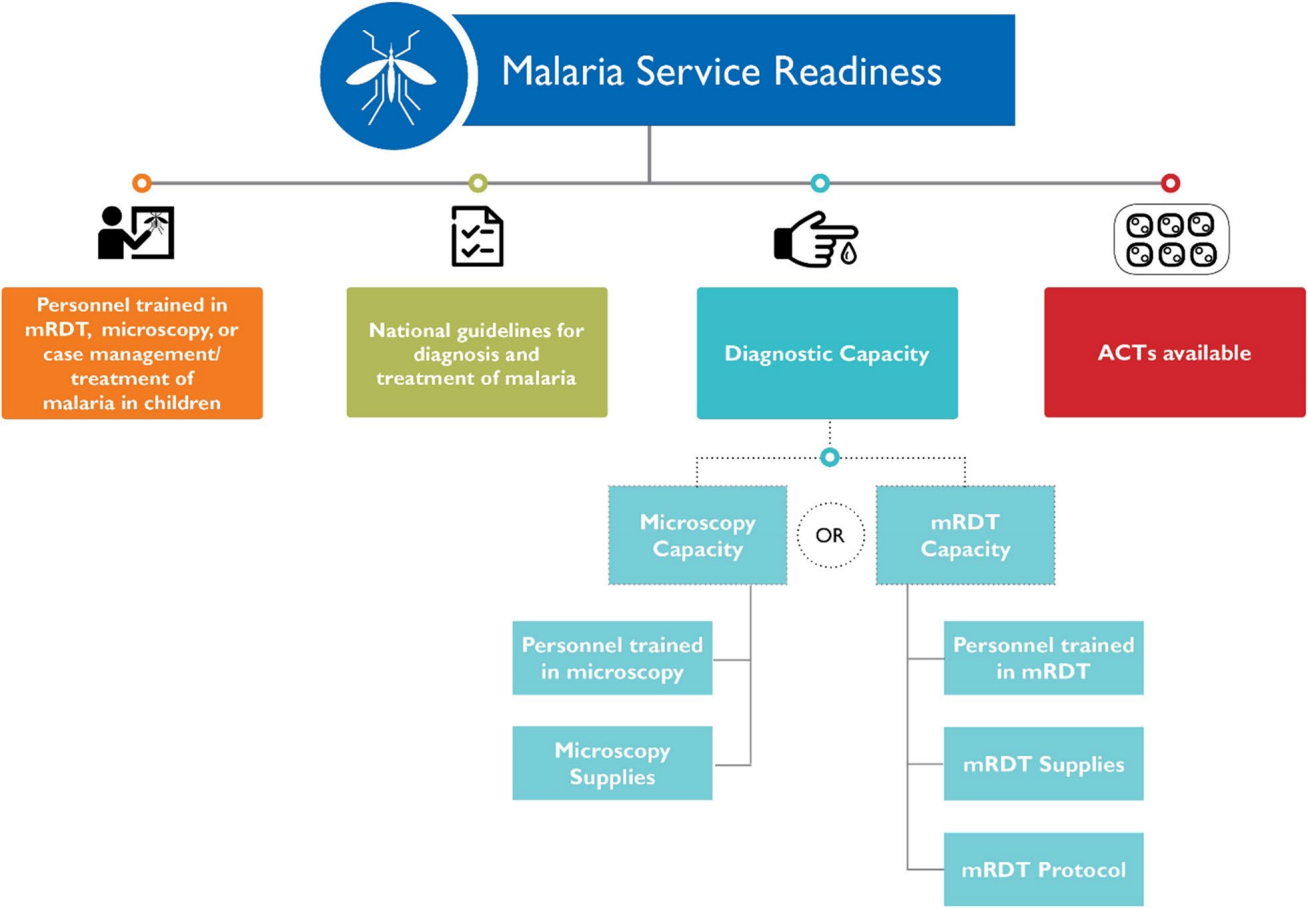
Components of malaria-service readiness

The facility-level indicator examined in this analysis is the proportion of facilities that are categorized as malaria-service ready as defined by the WHO Service Availability and Readiness Assessment (SARA) [41]. This indicator assesses the ability of a facility to provide sufficient care for the diagnosis and treatment of malaria. Facilities categorized as malaria-service ready are those with: (1) trained personnel (adequate training is defined as a facility having at least one provider of malaria services who reported having received in-service training during the 24 months before the survey in either RDT, microscopy or case management and treatment of malaria in children); (2) national guidelines for the diagnosis and treatment of malaria; (3) malaria diagnostic capacity with valid RDT tests or microscopy equipment (available equipment as well as staff trained in its use); and, (4) unexpired ACT medicine available on the day of the survey [41] (Fig. 1). As seen in Fig. 1, malaria diagnostic capacity is a composite indicator defined as a facility having microscopy diagnostic capacity or RDT diagnostic capacity. Microscopy diagnostic capacity is defined as a facility having a functioning microscope with glass slides and relevant stains, in addition to at least one health provider who received training on microscopy during the 24 months before the survey. Similarly, RDT diagnostic capacity is defined as a facility having unexpired malaria RDT kits, at least one health provider who received RDT training in the 24 months before the survey, and the facility having an instructional protocol for performing a RDT. It should be noted that the definition of malaria-service readiness is tailored towards non-severe paediatric malaria cases since it does not include the availability of pre-referral medication or severe malaria treatments (e.g., rectal artesunate or injectable anti-malarials for severe cases).

The SPA and DHS are independent of each other, meaning that there are no linkages between specific facilities listed in the SPA and DHS surveys. Without this linkage, it is not possible to know the service readiness of the exact facility where the child’s caregiver sought advice and treatment for their child with a fever. Since linkage of DHS and SPA surveys is not possible, this analysis provides an overview of the malaria service readiness (according to SPA surveys) of the specific facility categories where caregivers most frequently sought care for children under age 5 years with fever (according to the DHS surveys).

In order to examine the types of facilities where individuals tend to seek care and the malaria-service readiness of those facilities, the study needed to harmonize categories of facility types between the DHS and SPA surveys, to allow for comparisons between care seeking and provision of malaria services at specific facility types. Generally, the DHS questionnaire groups health facilities by public, religious, private, and other sources, while the SPA categorizes facilities by managing authority: government, private, non-governmental organization (NGO), religious (and facility level) hospital, health centre, dispensary, clinic. The facility types were harmonized (considering managing authority and facility level) between the DHS and SPA surveys in order to examine the types of facilities where individuals seek care and the malaria service readiness of those facilities (Table 1). Efforts were made to group facilities by facility level, although this was not possible in all cases due to sample size.

**Table 1:** Standardized health facilities

Standardized name	DHS facilities	SPA facilities
**Malawi**	**2015–2016 Malawi DHS**	**2013–2014 Malawi SPA**
Government hospital	Government hospital	Government/public: central hospital Government/public: district hospital Government/public: rural/community hospital Government/public: other hospital
Government health centre	Government health centre	Government/public: health centre
Government health post or mobile clinic	Government health post/outreach Government mobile clinic Government HSA	Government/public: maternity Government/public: dispensary Government/public: clinic Government/public: health post
Private facility	Private hospital/clinic Private pharmacy Private doctor Private mobile clinic Private fieldworker/CHW Other private medical sector	Private for profit: other hospital Private for profit: health centre Private for profit: maternity Private for profit: dispensary Private for profit: clinic NGO: other hospital NGO: health centre NGO: clinic Company: health centre Company: dispensary Company: clinic
Christian health association/mission/faith-based	CHAM/mission hospital CHAM/mission health centre	CHAM: rural/community hospital CHAM: other hospital CHAM: health centre CHAM: maternity CHAM: dispensary CHAM: clinic CHAM: health post Mission/faith-based (other than CHAM): Other hospital Mission/faith-based (other than CHAM): Clinic
Any other source	BLM Shop Traditional practitioner Market Itinerant drug seller Youth drop-in centre Other	No SPA equivalent
**Senegal**	**2016 Senegal Continuous DHS**	**2016 Senegal Continuous SPA**
Government hospital or health centre	Government hospital Government health centre	Government/public: hospital Government/public: health centre
Government health post or mobile clinic	Government health post Government mobile team	Government/public: clinic
Government health hut	Other public sector Country specific public sector	Government/public: case de santé
Private facility	Private hospital/clinic Private pharmacy Private community health worker Other private medical	NGO/private not for profit: hospital NGO/private not for profit: health center NGO/private not for profit: clinic Private for profit: hospital Private for profit: health centre Private for profit: clinic
Any other source	Shop Traditional practitioner Country-specific other sector Other	No SPA equivalent
**Tanzania**	**2015–2016 Tanzania DHS-MIS**	**2014–2015 Tanzania SPA**
Government hospital	National/zonal referral/specialized hospital Referral regional hospital Regional hospital District hospital	Government/public: national referral hospital Government/public: regional hospital Government/public: district hospital Government/public: district-designated hospital Government/public: other hospital Parastatal: regional hospital Parastatal: other hospital
Government health centre	Public health centre	Government/public: health centre Parastatal: health center
Government clinic/dispensary	Public clinic Public dispensary Public CHW	Government/public: clinic Government/public: dispensary Parastatal: dispensary
Private facility	Private hospital Private specialized hospital Private health centre Private clinic Private dispensary	Private: other hospital Private: health centre Private: clinic Private: dispensary
Mission/faith-based	Religious/voluntary referral spec. hospital religious/voluntary hospital religious/voluntary district hospital religious/voluntary health centre religious/voluntary clinic religious/voluntary dispensary	Mission/faith-based: national referral Mission/faith-based: regional hospital Mission/faith-based: district hospital Mission/faith-based: district-designated hospital Mission/faith-based: other hospital Mission/faith-based: health centre Mission/faith-based: clinic Mission/faith-based: dispensary
Any other source	Pharmacy ADDO NGO Other	No SPA equivalent

*HSA* Health Surveillance Assistants, *CHAM* Christian Health Association of Malawi, *BLM* Banja La Mtsogolo (program established by Marie Stopes International), *ADDO* Accredited Drug Dispensing Outlet

One limitation of the SPA survey is that it only captures the services provided by formal-sector health facilities. SPA surveys do not include some private health care providers (individual doctors operating outside sampled facilities, private pharmacies), community-based care (community health workers (CHWs) who provide integrated community case management (iCCM)), or those in the informal health care sector (traditional healers, markets, shops). Although the DHS questionnaire includes options for private healthcare providers and informal healthcare providers, it is impossible to compare data on these facilities with the data in the SPA. In this analysis, therefore, private healthcare providers and providers in the informal healthcare sectors are identified as ‘any other source’.

The study includes a country-level descriptive analysis with 95% confidence intervals of both DHS and SPA data. Estimates from both DHS and SPA surveys were adjusted for complex survey design and relevant sample weights. All analyses were conducted using Stata15 (StataCorp LP, College Station, USA).

## Results

### Malawi

Among facilities sampled in the 2013–2014 Malawi SPA that offered malaria diagnosis/treatment services or curative care for sick children, 25% had all the components to be considered malaria-service ready (Table 2). Individual components of malaria-service readiness ranged from 92% of facilities having ACT available to 34% having diagnostic capacity. In the Malawi 2015–2016 DHS, advice and treatment was sought for 67% of the children under 5 years old with fever in the 2 weeks before the survey. Among febrile children for whom advice or treatment was sought, the most used source for care was government health centres (39%). Malaria-service readiness in government health centres was 32%, slightly higher than the average of 25% among all facilities. Disaggregating service readiness into individual components, government health facilities were performing well in several areas: 99% had ACT available, and 71% had national guidelines for the diagnosis and treatment of malaria available at the facility. The major gap in service readiness among government health centres in Malawi was a low level of diagnostic capacity (41%). Examining the components of diagnostic capacity among government health centres reveals that 3% had microscopy diagnostic capacity and 41% had RDT diagnostic capacity. While most facilities had RDT supplies (95%) only 61% had at least one health provider who received RDT training in the 24 months before the survey (Table 3).

**Table 2:** Source of advice or treatment for children with fever and components of malaria service readiness by facilities in Malawi, Senegal and Tanzania

Facility type	DHS Survey Data		SPA Survey Data					
	% for whom advice or treatment was sought	Number of children under 5 years with a fever	Among facilities offering malaria diagnosis/treatment services or curative sick child care services, percentage with					
			Personnel trained in RDT, microscopy or case management treatment of malaria in children	National guidelines for diagnosis and treatment of malaria	Diagnostic capacity^a^	ACT available	Malaria service readiness index	Total number of facilities
**Malawi**	**2015–2016 Malawi DHS**		**2013–2014 Malawi SPA**					
Government hospital	7.8 [6.5–9.2]	4774	83.9 [70.9–91.7]	82.0 [68.9–90.4]	75.9 [62.1–85.8]	100.0	59.9 [45.9–72.5]	48
Government health centre	38.6 [36.4–40.9]	4774	65.4 [60.2–70.2]	71.2 [66.2–75.7]	41.4 [36.4–46.7]	99.2 [97.7–99.9]	32.0 [27.3–37.1]	338
Government health post or mobile clinic	6.9 [5.8–8.3]	4774	40.9 [29.8–53.2]	60.4 [48.2–71.5]	19.6 [11.7–30.9]	80.8 [69.2–88.7]	14.9 [8.2–25.7]	68
Private facility	4.9 [3.8–6.2]	4774	38.8 [33.6–44.2]	50.8 [45.4–56.3]	19.1 [15.2–23.8]	81.5 [76.9–85.4]	11.7 [8.6–15.7]	330
Christian health association/mission/faith-based	3.9 [3.1–4.9]	4774	57.8 [50.1–65.1]	70.4 [63–76.9]	43.3 [36.0–51.0]	98.8 [95.3–99.7]	32.5 [25.8–40.0]	162
Any other source	5.9 [5.0–6.9]	4774	n/a	n/a	n/a	n/a	n/a	n/a
Total	67.1 [65.1–69.0]	4774	54.0 [50.8–57.1]	63.7 [60.6–66.8]	34.2 [31.2–37.2]	91.8 [89.8–93.4]	25.2 [22.5–28.0]	947
**Senegal**	**2016 Senegal Continuous DHS**		**2016 Senegal Continuous DHS**					
Government hospital or health centre	4.6 [3.0–7.1]	692	86.2 [74.4–93.1]	91.1 [79.8–96.4]	81.9 [69.8–89.8]	89.8 [78.4–95.5]	77.0 [64.4–86.0]	29
Government health post or mobile clinic	32.3 [27.0–38.1]	692	96.2 [92.1–98.2]	94.1 [89.8–96.7]	73.8 [67.3–79.4]	96.0 [92.4–97.9]	68.8 [62.1–74.8]	252
Government health hut	3.4 [2.0–5.7]	692	84.2 [69.5–92.5]	76.0 [60.5–86.7]	27.6 [17.7–40.2]	63.0 [48–75.8]	20.9 [12.2–33.3]	85
Private facility	8.6 [5.3–13.9]	692	64.5 [51.7–75.5]	71.9 [60–81.4]	41.6 [30.3–53.9]	48.5 [36.5–60.6]	26.3 [16.9–38.5]	70
Any other source	2.8 [1.5–4.9]	692	n/a	n/a	n/a	n/a	n/a	n/a
Total	49.8 [45.1–54.6]	692	88.1 [83.9–91.3]	86.8 [82.4–90.2]	60.1 [54.7–65.3]	81.5 [76.7–85.5]	53.2 [47.8–58.4]	436
**Tanzania**	**2015–2016 Tanzania DHS-MIS**		**2014–2015 Tanzania SPA**					
Government hospital	3.8 [2.6–5.3]	1706	66.5 [48.9–80.5]	47.8 [35.1–60.8]	61.7 [45.6–75.6]	91.0 [84.5–95.0]	33.3 [23.7–44.4]	24
Government health centre	5.8 [4.4–7.7]	1706	73.5 [67.8–78.6]	61.0 [54.8–66.9]	57.4 [51.2–63.4]	94.5 [91.2–96.6]	35.3 [29.6–41.4]	88
Government clinic/dispensary	18.8 [15.8–22.2]	1706	46.8 [41.4–52.2]	64.9 [59.5–70.0]	26.5 [22.1–31.5]	94.2 [91.0–96.2]	18.5 [14.8–23.0]	761
Private facility	7.7 [6.1–9.7]	1706	27.5 [18.4–38.8]	42.5 [31.0–54.9]	20.9 [13.0–31.8]	73.7 [61.3–83.2]	10.9 [5.2–21.4]	156
Mission/faith-based	3.9 [2.9–5.3]	1706	47.7 [36.4–59.3]	60.4 [48.6–71.1]	20.5 [13.9–29.2]	84.1 [73.1–91.2]	12.2 [7.5–19.1]	147
Any other source	54.3 [50.7–57.8]	1706	n/a	n/a	n/a	n/a	n/a	n/a
Total	81.3 [78.5–83.6]	1706	46.7 [42.6–50.9]	60.7 [56.5–64.8]	28.1 [24.7–31.7]	90.2 [87.3–92.4]	18.3 [15.5–21.5]	1177

See Table 3 for more details on the components of diagnostic capacity

^a^Diagnostic capacity is defined as a facility having microscopy diagnostic capacity or RDT diagnostic capacity

**Table 3:** Components of diagnostic capacity by facilities in Malawi, Senegal and Tanzania

Facility type	Among facilities offering malaria diagnosis/treatment services or curative sick child care services, percentage with								
	Components of microscopy diagnostic capacity			Components of RDT diagnostic capacity			RDT diagnostic capacity^b^	Malaria diagnostic capacity^c^	Total number of facilities
	Personnel trained in microscopy	Microscopy supplies	Microscopy diagnostic capacity^a^	Personnel trained in RDT	RDT supplies	RDT protocol			
**Malawi**	**2013–2014 Malawi SPA**								
Government hospital	71.9 [57.9–82.6]	60.1 [46.0–72.6]	39.9 [27.4–54]	81.9 [68.7–90.3]	98 [87.1–99.7]	88 [75.8–94.5]	71.9 [57.9–82.6]	75.9 [62.1–85.8]	48
Government health centre	48.1 [42.9–53.4]	4.6 [2.8–7.4]	2.9 [1.5–5.3]	60.5 [55.2–65.5]	94.8 [91.9–96.7]	68.9 [63.8–73.5]	40.9 [35.8–46.1]	41.4 [36.4–46.7]	338
Government health post or mobile clinic	25.7 [16.6–37.6]	3.0 [0.7–11.1]	1.4 [0.2–9.5]	36.1 [25.4–48.3]	79.6 [68–87.8]	52.8 [40.8–64.6]	19.6 [11.7–30.9]	19.6 [11.7–30.9]	68
Private facility	28.3 [23.6–33.5]	12.6 [9.4–16.7]	4.0 [2.3–6.8]	34.4 [29.4–39.8]	75.6 [70.6–80]	45.6 [40.3–51.1]	17.6 [13.8–22.2]	19.1 [15.2–23.8]	330
Christian health association/mission/ faith-based	43.9 [36.5–51.5]	24.7 [18.7–31.8]	15.0 [10.3–21.3]	55.3 [47.7–62.7]	97.5 [93.6–99.1]	68.1 [60.6–74.7]	38.5 [31.4–46.1]	43.3 [36.0–51.0]	162
Total	40.1 [37–43.2]	13.5 [11.5–15.9]	7.1 [5.7–8.9]	49.8 [46.7–53]	87.6 [85.3–89.6]	60.5 [57.3–63.5]	32.4 [29.5–35.4]	34.2 [31.2–37.2]	947
**Senegal**	**2016 Senegal Continuous DHS**								
Government hospital or health centre	86.2 [74.4–93.1]	80.7 [68.4–88.9]	74.3 [61.6–83.9]	62.0 [48.9–73.5]	98.4 [89.2–99.8]	92.6 [81.4–97.3]	60.3 [47.3–72.1]	81.9 [69.8–89.8]	29
Government health post or mobile clinic	96.2 [92.1–98.2]	1.2 [0.4–4]	1.2 [0.4–4]	80.5 [74.4–85.4]	98.5 [95.3–99.5]	90.9 [86.1–94.2]	73.3 [66.8–79]	73.8 [67.3–79.4]	252
Government health hut	84.2 [69.5–92.5]	0.0 [0.0–0.0]	0.0 [0.0–0.0]	41.7 [29.2–55.4]	74.5 [57.7–86.2]	60.6 [45.9–73.6]	27.6 [17.7–40.2]	27.6 [17.7–40.2]	85
Private facility	64.5 [51.7–75.5]	26.8 [17.4–39]	20.2 [12.1–31.8]	36.3 [25.7–48.6]	74.9 [62.5–84.2]	68.6 [56.0–78.9]	27.9 [18.7–39.5]	41.6 [30.3–53.9]	70
Total	88.1 [83.9–91.3]	10.4 [8–13.4]	8.9 [6.8–11.6]	64.6 [59.2–69.6]	90 [85.4–93.3]	81.5 [76.6–85.6]	56.2 [50.9–61.5]	60.1 [54.7–65.3]	436
**Tanzania**	**2014–15 Tanzania SPA**								
Government hospital	61.7 [45.5–75.6]	55.4 [41.0–69.0]	43.0 [31.4–55.5]	56.0 [41.4–69.7]	93.8 [88.2–96.9]	88.9 [81.9–93.4]	50.5 [37.2–63.8]	61.7 [45.6–75.6]	24
Government health centre	65.7 [59.7–71.3]	44.7 [38.7–50.9]	33.1 [27.5–39.1]	65.9 [59.9–71.5]	92.5 [88.5–95.2]	79.7 [74.3–84.2]	49.7 [43.6–55.8]	57.4 [51.2–63.4]	88
Government clinic/dispensary	33.5 [28.6–38.7]	4.9 [2.9–7.9]	0.9 [0.3–2.5]	40.8 [35.6–46.2]	83.8 [79.4–87.5]	64.7 [59.3–69.7]	26.4 [21.9–31.3]	26.5 [22.1–31.5]	761
Private facility	24.7 [16.0–36.1]	38.5 [27.4–50.9]	7.6 [4–14]	25.7 [16.9–37.1]	63.1 [50.6–74]	54.8 [42.5–66.5]	18.2 [10.7–29.2]	20.9 [13.0–31.8]	156
Mission/faith-based	38.1 [27.9–49.5]	37.9 [27.5–49.6]	12.6 [7.7–19.8]	35.7 [25.9–46.9]	72.4 [60.4–81.9]	54.1 [42.4–65.4]	16.9 [10.9–25.2]	20.5 [13.9–29.2]	147
Total	35.9 [32.1–39.9]	17.5 [14.7–20.7]	6.6 [5.4–8]	40.4 [36.4–44.5]	80.5 [76.9–83.7]	63.7 [59.5–67.7]	26.4 [23–30]	28.1 [24.7–31.7]	1177

^a^Microscopy diagnostic capacity is defined as a facility having a functioning microscope with glass slides and relevant stains, in addition to at least one health provider who has received training on microscopy during the 24 months before the survey

^b^RDT diagnostic capacity is defined as a facility having unexpired malaria RDT kits, at least one health provider who received RDT training in the 24 months before the survey, and the facility having an instructional protocol for performing a RDT

^c^Diagnostic capacity is defined as a facility having microscopy diagnostic capacity or RDT diagnostic capacity

### Senegal

Overall, 53% of facilities in Senegal sampled in the 2016 SPA that offered malaria diagnosis/treatment services or curative care for sick children had all the components to be considered malaria-service ready. Among the individual components of malaria-service readiness, Senegal performed highest in having personnel trained in malaria diagnosis (88%) and posted guidelines in the facility (87%). Senegal’s facilities performed less well with diagnostic capacity (60%), however. The corresponding 2016 Senegal DHS survey showed that advice or treatment was sought for 50% of the children with fever in the 2 weeks before the survey. The most commonly used source of advice or treatment for children with fever was primary-level facilities (government health post or mobile clinic), at 32%. Among government health posts or mobile clinics in Senegal, 69% met the criteria to be considered malaria-service ready, substantially higher than the 53% average among all facilities. Among the primary-level facilities, 96% had ACT medicines available and personnel trained in malaria diagnosis or treatment, and 94% had national guidelines for diagnosis and treatment of malaria, while 74% had diagnostic capacity (Table 2). Most government health posts or mobile clinics had RDT diagnostic capacity (73%) and 99% of government health posts or mobile clinics had RDT supplies. Very few government health posts or mobile clinics had microscopy diagnostic capacity (1%) (Table 3).

### Tanzania

On average, facilities in Tanzania were least ready to provide malaria services; only 18% of facilities sampled in the 2014–2015 SPA had all four service readiness components, highest for the component on availability of ACT (90%). Tanzanian facilities performed poorly in diagnostic capacity (28%). In the Tanzania 2015–2016 DHS-MIS survey, advice or treatment was sought for 81% of children under 5 years old with fever in the 2 weeks before the survey. What differentiates Tanzania from other two countries studied is that for 54% of febrile children for whom advice or treatment was sought, it was from a source not sampled in the SPA survey. For Tanzania this included ADDOs and other pharmacies. However, among the sources sampled in the SPA survey the highest proportion (19%) of febrile children were taken to a government clinic/dispensary. Among government clinics/dispensaries, only 19% were malaria-service ready, much lower than among health centres and hospitals. Among government clinics/dispensaries, 94% had ACT medicines available, 65% had national guidelines for diagnosis and treatment of malaria, and 47% had personnel trained in malaria diagnosis or treatment. The major barrier to service readiness for government clinics/dispensaries, as for sampled facilities overall in Tanzania, was low diagnostic capacity (27%) (Table 2). Twenty-seven per cent of government clinics/dispensaries had RDT diagnostic capacity and 1% had microscopy diagnostic capacity. In examining the components of RDT capacity in government clinics/dispensaries, 41% had at least one health provider who received RDT training in the 24 months before the survey and 65% had a RDT protocol posted in the facility. Availability of RDTs do not appear to be a limiting factor in that 84% of facilities had RDT supplies available on the day of the survey (Table 3).

## Discussion

The percentage of facilities classified as malaria-service ready ranged from 18% in Tanzania and 25% in Malawi to 53% in Senegal. In all three countries, primary healthcare facilities (health centre/health post/health clinic) were the most used type of facility for care seeking for febrile children. Among these primary healthcare facilities, only 69% in Senegal, 32% in Malawi and 19% in Tanzania were considered as malaria-service ready, with all four required components of malaria-service readiness, although they performed better in individual components. Among the four components of malaria-service readiness in the facilities most visited by febrile children, diagnostic capacity (comprising supplies and trained personnel) was the weakest component in all three countries, due to the low availability of trained personnel. The next weakest component was personnel trained in RDT, microscopy or case management/treatment of malaria in children for Tanzania and Malawi, and national guidelines posted for diagnosis and treatment of malaria in Senegal. These results are similar to Lee et al. who examined malaria-service readiness in relationship to malaria endemicity in Kenya, Namibia and Senegal [17] and to Dolan et al. who examined malaria-service readiness as it pertains to health aid projects in Malawi [42]. Both studies also identified a lack of malaria guidelines and adequately trained staff as reasons for lower malaria service readiness in facilities.

More emphasis on training personnel would achieve the greatest improvements in malaria-service readiness of facilities most frequented by febrile children in the studied countries. Having trained personnel is not only one of the four components of malaria-service readiness but also a sub-component of diagnostic capacity. Ongoing training for service providers can ensure that providers continue to learn about the most recent developments in malaria care. In the SPA, adequate training is defined as a facility having at least one provider of malaria services who reported receiving in-service training during the 24 months before the survey. The training must include structured sessions and does not include individual instruction a provider might have received during routine supervision. Although personnel might have received training in the past (more than 24 months before to the survey) malaria policies are continually changing. There can be repercussions on the quality of diagnosis and treatment in facilities that do not have at least one provider trained in updated policies.

To improve malaria-service readiness, governments should focus on in-service training, training updates, or refresher training on the performance of malaria microscopy or RDTs, as well as correct case management procedures. This would improve not only the trained provider component of malaria-service readiness but also diagnostic capacity. While previous studies have shown mixed conclusions around the impact of large-scale investments in in-service training [43–46], it is important to offer in-service training not just once but on a regular basis and updated based on changing policies. Additionally, training that targets smaller groups, focus on a single topic and incorporate principles of adult learning have been shown to produce better results in improving performance among health workers [47].

All three countries reported high levels of availability of ACT medicine in the facilities most used by children with fever. The universal and continuous availability of anti-malarial drugs is a critical component in the delivery of malaria treatment in health facilities. Unexpired ACT medicine was available in 99% of facilities most frequented by febrile children in Malawi, 96% of facilities in Senegal and 94% of facilities in Tanzania. This finding is encouraging, because children who are positive for malaria can be treated with ACT within the facility where treatment is sought. However, this successful component of malaria-service readiness must be maintained in the future.

One important limitation of this study is the lack of linkages (SPA and DHS are independent surveys fielded at different times) between specific facilities in the DHS and SPA surveys. Without this linkage, the service readiness of the exact facilities where the children’s caregivers sought advice and treatment for children with a fever is unknown. In this analysis the SPA surveys provided information on the malaria-service readiness of facilities, while the DHS surveys provided information on population-level utilization of malaria services in children under 5 years old with fever. Linking these two types of data sources can be challenging, because the SPA and DHS are independent surveys and not designed to be linked. In addition, publicly available geolocations of DHS clusters are displaced to protect confidentiality of the participants.

Another limitation is that, because the SPA covers only formal health facilities, the analysis does not include the service readiness of informal health services or private healthcare providers who operate outside a formal facility. There is evidence that use of private healthcare providers and informal healthcare providers, such as pharmacies for the disbursement of anti-malarial drugs, could be more widespread than use of formal facilities. In the 2015–2016 Tanzania DHS-MIS, for example, 54% of children who sought advice or treatment for fever were taken to a location categorized as an ‘other source’. The primary reason that smaller private providers, informal healthcare providers, or CHWs who provide iCCM are not included in a SPA survey is the absence of an accurate sampling frame for these providers. Since many of these providers are not well documented or do not have the proper accreditation, the country’s Ministry of Health may not have a complete listing that could serve as the basis for a sampling frame. Further research is needed on the readiness and quality of services provided by informal providers in settings where the informal sector is widely used for malaria diagnosis and treatment.

The definition of malaria-service readiness is also problematic in that it is very hard to achieve, especially for lower-level health facilities. The malaria-service readiness index has many components and may not always accurately measure true readiness. For example, if a facility has all the components of RDT diagnostic capacity but do not have RDT protocol (training manual, poster or job aid for using RDT) observed in the facility, the facility does not have RDT diagnostic capacity and in turn is not considered to be malaria-service ready. Additionally, the definition of in-service training for malaria-service readiness is very specific in stating that the training must include structured sessions and does not include individual instruction a provider might have received during routine supervision. Over time many organizations are choosing to support training of frontline health workers through supervision, not through classroom training. If a facility has been supported by an organization that funds training through routine supervision instead of structured classroom settings, this facility is no longer classified as malaria-service ready since there will be no provider in the facility trained in structured sessions. These are limitations that should be considered in the future for the WHO definition of malaria service readiness.

It should also be noted that the malaria-service readiness definition used here is based on guidelines for the treatment of malaria rather than integrated treatment protocols for sick children (it does not include availability of antibiotics, for example), which may limit the interpretation of these findings.

Lastly, in harmonizing facility types between the DHS and SPA surveys, several types of health facilities had to be grouped together to provide a large enough sample size for stratification. In Senegal, for example, due to low numbers of individuals seeking care for fever at government hospitals, government hospitals and health centres were combined to generate an adequate sample size for analysis. An assumption was made that the level of service readiness is the same among the combined facilities, which is not necessarily the case.

## Conclusions

This study investigated the utilization and provision of malaria services by examining data on the source of advice or treatment in children under 5 years old with fever from the household-based DHS surveys and data on provision of care from the facility-based SPA surveys from Malawi, Senegal and Tanzania. The analysis highlights the need for improving the malaria-service readiness of facilities in all three countries. More effort should be focused on facilities that are commonly used for care and treatment of fever, especially in the areas of malaria diagnostic capacity and provider training. It is essential for policymakers to consider the malaria-service readiness of primary-level healthcare facilities when allocating resources and training, particularly in limited-resource settings, to ensure that the points of care that are most used are properly equipped to provide diagnosis and treatment for malaria.

## Data Availability

The datasets analysed during the current study are available from The DHS Program web site, http://www.dhsprogram.com.

## Abbreviations

ACT: artemisinin-based combination therapy
ADDO: accredited drug dispensing outlet
CHAM: Christian Health Association of Malawi
CHW: Community Health Worker
DHS: Demographic and Health Survey
FBOs: Faith-based Organizations
iCCM: integrated community case management
IMCI: integrated management of childhood illness
RDT: malaria rapid diagnostic test
MoHCDEC: Ministry of Health, Community Development, Gender, Elderly and Children
MIS: Malaria Indicator Survey
NGO: Non-Governmental Organization
PHCCs: primary health care cottages
PHCUs: primary health care units
RBM: roll back malaria
SPA: Service Provision Assessment
WHO: World Health Organization
